# Assessment of measles immunity among infants in Maputo City, Mozambique

**DOI:** 10.1186/1471-2458-8-386

**Published:** 2008-11-12

**Authors:** Jagrati V Jani, Carol Holm-Hansen, Tufária Mussá, Arlinda Zango, Ivan Manhiça, Gunnar Bjune, Ilesh V Jani

**Affiliations:** 1 Department of Immunology, Instituto Nacional de Saúde, Mozambique; 2 Department of General Practice and Community Medicine, University of Oslo, Norway; 3 Division of Infectious Disease Control, Norwegian Institute of Public Health, Norway

## Abstract

**Background:**

The optimum age for measles vaccination varies from country to country and thus a standardized vaccination schedule is controversial. While the increase in measles vaccination coverage has produced significant changes in the epidemiology of infection, vaccination schedules have not been adjusted. Instead, measures to cut wild-type virus transmission through mass vaccination campaigns have been instituted. This study estimates the presence of measles antibodies among six- and nine-month-old children and assesses the current vaccination seroconversion by using a non invasive method in Maputo City, Mozambique.

**Methods:**

Six- and nine-month old children and their mothers were screened in a cross-sectional study for measles-specific antibodies in oral fluid. All vaccinated children were invited for a follow-up visit 15 days after immunization to assess seroconversion.

**Results:**

82.4% of the children lost maternal antibodies by six months. Most children were antibody-positive post-vaccination at nine months, although 30.5 % of nine month old children had antibodies in oral fluid before vaccination. We suggest that these pre-vaccination antibodies are due to contact with wild-type of measles virus. The observed seroconversion rate after vaccination was 84.2%.

**Conclusion:**

These data indicate a need to re-evaluate the effectiveness of the measles immunization policy in the current epidemiological scenario.

## Background

Measles elimination is one of the major global public health priorities [1]. While half of the world is close to eliminating measles, many countries in Sub-Saharan Africa (SSA) are still struggling to control the disease [2]. In Mozambique, the measles vaccine was introduced in 1979 through an immunization campaign that targeted children between the ages of six months to three years. The Expanded Programme on Immunization (EPI) started in 1981 [3]. Since then, the main intervention for measles control in Mozambique has been the routine administration of a single dose of measles vaccine. Additionally, in 2005 the Ministry of Health (MoH) of Mozambique adopted fourth yearly national vaccination campaigns targeting children younger than 14 years. Despite these efforts to increase vaccination coverage, measles epidemics continue to occur periodically [4].

The increase in measles vaccination coverage in the developing world has produced significant changes in the epidemiology of the infection [5], such as a shift to a higher incidence of measles in older children and young adults [6]. Moreover, a significant proportion of women at the reproductive age may now have measles immunity as a result of vaccination. When compared to women who had natural infection, vaccinated women are expected to have lower titres of measles antibodies and give birth to offspring that remain passively protected against the measles virus for a shorter period of time [7,8].

In most SSA countries, the change in the epidemiology of infection has not been accompanied by an adjustment in vaccination schedules. Instead, measures to cut wild- virus transmission through mass vaccination campaigns have been instituted [9-11]. In parallel, some countries have started to report the consequences of mass vaccination on the passive immunity against measles in infants and to re-evaluate the efficacy of current vaccination schedules in light of the new epidemiological scenario [12]. The data obtained from these studies will prove crucial in designing public health interventions for measles control and elimination [13].

Epidemiological studies on wild-type-virus or vaccine-induced immunity have classically been performed through the detection of measles-specific IgG and IgM in serum or plasma [14,15]. The use of these biological specimens under field conditions in resource-poor-settings has posed critical logistical challenges to the implementation and success of epidemiological studies and surveys [15]. Hence, the detection of measles-specific antibodies in oral fluid samples has been recommended as a safe, effective and non-invasive alternative to serum and/or plasma for the diagnosis of disease and immune surveillance in Africa [16,17].

In this study, the detection of measles-specific antibodies in oral fluid was employed to: 1) assess the level of passive immunity against measles among six- and nine-month-old infants, and 2) evaluate the immune response against measles vaccine in nine-month-old infants.

## Methods

### Population and study design

This study was conducted in Maputo City, the capital of Mozambique, which has an estimated population of 1.5 million people. Health services in Maputo are organized in three districts, each served by several health-centres and a general hospital. Health-centres offer a free health program for all children under the age of five-years. The program includes immunization, growth monitoring and nutritional rehabilitation.

Subjects were recruited at the Xipamanine health-centre and the 1° de Junho health-centre located in the urban districts number four and two of Maputo City. The urban districts number four and two have 300,703 and 534,744 inhabitants and reported measles vaccine coverage of 80% and 83%, respectively (district midterm reports to MoH, unpublished document, 2005).

The study was performed between June and September 2005, just before the 2005 measles national mass vaccination campaign. According to the EPI schedule in Mozambique, children are routinely immunized with a single dose of standard titre measles vaccine at nine months of age. At the time when the study was conducted, the measles vaccine in use was based on Edmonston-Zagreb strain (E-Z). Mothers of six-month-old children coming for growth monitoring and of nine-month-old children visiting the health-centre for measles immunization were invited to participate in the study. All children and their respective mothers were screened in a cross-sectional study for measles-specific antibodies in oral fluid. Additionally, all children at nine-months of age were invited to donate a second specimen of oral fluid during a follow-up visit 15 days after vaccination.

This study was approved by the Mozambican Health Bioethics Committee. Informed consent was obtained by a signature or finger print from mothers after explaining the project aims, the procedures for data and specimen collection, and the need to return for post-vaccination control among those vaccinated.

### Data collection and definitions

Data was collected using a structured questionnaire. The age of each child was calculated from the date of birth recorded on the Road-to-Health Card. The age of the mother was based on her verbal statement. A birth was considered to be *premature *if the baby was born at less than 37 weeks of gestation. This information was collected from the mother's Pregnancy-Monitoring Card. A baby with a birth weight less than 2,500 grams was defined as having *low-birth-weight*. This information was collected from the Road-to-Health Card.

On admission, the children were weighed (grams) and measured (centimetres). The anthropometric indices weight-for-age, height-for-age and weight-for-height were compared with mean Z-scores to access the nutritional status of the children. Minus two Z-scores were used as cut-off values for low height-for-age, weight-for-age and weight-for-height indices.

The immunization status of the mothers was confirmed by the Road-to-Health Card when possible. A verbal history of immunization of the mother with no card for confirmation was categorized as a "history of immunization". Mothers with no knowledge of their past immunization were classified as having an "unknown" status. A past history of measles disease in the infant and in the mother was collected by verbal history using the World Health Organization case definition [18]. The mother's reproductive history was collected from the last gestation Pregnancy-Monitoring Card retained by the mother. Verbal information was not considered.

### Sample collection and processing

Oral fluid was collected using the OraSure device (OraSure Technologies, Bethlehem, PA, USA). The collection device consists of a 3 cm × 1 cm flat pad of absorbent material supported by a 10 cm plastic stick. This device is supplied with a tube containing transport buffer and a preservative [19,20]. The absorbent pad was moved gently 4–10 times along the gums and left stationary between the lower gum and buccal membrane for a minimum of two minutes or until the pad was saturated with oral fluid. Thereafter, the collection device was placed in the pre-coded tube containing the buffer. The pads with oral fluid samples were transported to the laboratory at the Instituto Nacional de Saúde every day. There, tubes were centrifuged at 2000 rpm for five minutes. The fluid was then transferred into a screw-capped vial and stored at -20°C until testing.

### Screening method

Oral fluid specimens were screened for measles-specific IgG and IgM using the MicroImmune^® ^test (MicroImmune Ltd, UK). Both assays are capture EIAs and clasiify antibody status as positive, negative and borderline. The IgM test has a reported sensitivity and specificity of 100.0% (95% CI 85.2–100.0) and 96.6% (95% CI 90.7–99.3), respectively [17]. The sensitivity and specificity of the IgG assay are 97.5% (95% CI 96.1–98.3) and 86.7% (95% CI 78.4–91.5), respectively [16,21].

### Data analysis

Proportions and chi-square were used as a statistical test at a 5% significance level. The differences in mothers' ages were assessed by one way analysis of variance.

Statistical procedures were performed using the Statistical Package for the Social Sciences (SPSS) version 15.0. Seroconversion was expressed as the proportion of seronegative children before vaccination who became positive for measles-specific antibody (IgM and IgG) 15 days after vaccination. The statistical difference in the proportions was compared using chi-square test with a 5% significance level. Samples with borderline results were excluded from this analysis.

## Results

A total of 211 six-month-old children and 301 nine-month-old infants, and their respective mothers, were enrolled in the study. Of all nine-month-old infants invited for the follow-up visit, 198 (65.8%) returned for antibody testing 15 days after immunization.

### Measles-specific antibodies in six-month-old infants

Only 26 (12.3%) out of 211 six-month-old infants tested positive for measles IgG, while 174 (82.4%) tested negative for IgG and 11 (5.2%) were borderline (Table 1). Two out of the 26 infants who tested positive for IgG also tested positive for IgM, indicating a possible recent exposure to the virus.

**Table 1:** IgG In oral fluid among six- and nine-month-old infants before receiving standard dose Edmonston-Zagreb vaccine

**Mothers**		**Six-month-old infants**						**Nine-month-old infants**						
**IgG**	Age (yrs)*	**Pos**	% (95%CI)	**Neg**	% (95%CI)	**BL**	% (95%CI)	**Pos**	% (95%CI)	**Neg**	% (95%CI)	**BL**	% (95%CI)	Total
	
**Pos**	24	23	14.4 (9.8–20.7)	128	80.5 (73.3–86.1)	8	5 (2.3–10)	78	31.9 (26.2–38.2)	153	62.7 (56.2–68.7)	13	5.3 (2.9–9.1)	403
**Neg**	22	3	7.3 (2.5–19.4)	35	85.3 (70.1–93.9)	3	7.3 (1.9–21)	9	21.4 (11.7–35.9)	32	76.1 (61.4–86.5)	1	2.3 (0.4–12.3)	83
**BL**	23	0	-	11	100 (67.8–100)	0	-	3	33.3 (12.1–64.5)	6	66.6 (35.4–87.9)	0	-	20
Missing														6

***Total***		***26***		***174***		***11***		***90***		***191***		***14***		***512***

*median value

Pos – Positive; Neg – Negative, BL – Borderline value

Twenty three (88.4%) out of the 26 infants positive for measles IgG were born to mothers with measles IgG in oral fluid. The remaining three infants (11.5%) were born to IgG-negative mothers, suggesting that these infants had been in contact with the measles virus. All these three infants were IgM-negative.

None of the children had active clinical measles, a past history of measles or a known contact with anybody in the family or living in the neighbourhood with active measles. The mothers' ages ranged from 17–41 years for measles IgG-positive infants, 16–45 years for measles IgG-negative infants and 16–37 years for the borderline infants.

### Measles-specific antibodies in nine-month-old infants

Among the 301 nine-month-old infants included in the study, six children were not tested due to lack of a sufficient volume of oral fluid. Of the 295 children that were tested, a surprisingly high number 90 (30.5%) were positive for measles-specific IgG, while 191 (64.7%) tested negative for IgG and 14 (4.7%) were borderline. Among the 90 IgG-positive infants, 79 had sufficient quantity of oral fluid for IgM testing. Five of the tested specimens (6.3%) were simultaneously positive for IgM and IgG, suggesting recent contact with the measles virus.

Seventy eight (86.6%) of the 90 infants positive for measles IgG were born to mothers with measles IgG in oral fluid (Table 1). However, nine (10.0%) of the infants were born to IgG-negative mothers. From these nine infants, five were IgM-positive. Only one of these five children was exposed to a family member (an older sister) with measles. With this exception, none of the other children had active clinical measles, a past history of measles or a known contact with anybody from the family or living in the neighbourhood with active measles.

The mothers' ages ranged from 16–41 years for measles IgG-positive infants, 16–44 years for measles IgG-negative infants and 18–42 years for the borderline infants.

### Factors associated with the presence of measles-specific antibodies in six- and nine-month-old infants

#### Age of the mother

The mean age for mothers included in the study was 25 years (range 16–45 years). None of the mothers could document their vaccination status. The majority of the women (n = 345, 67.3%; 95% CI 63.3–71.4) were not sure if they had been vaccinated against measles during childhood, 68 (13.2%; 95% CI 10.3–16.2) said that they were vaccinated against measles and 99 (19.3%; 95% CI 15.9–22.8) answered that they had never been vaccinated against measles at any point in their lives.

The mothers were divided in two groups by age criteria: a group comprising women 29 years of age and older that probably had acquired measles immunity through natural infection, and a group including women 28 years of age and younger that probably had measles vaccine-induced immunity. The prevalence of measles IgG in these groups of mothers was 89.2% (95% CI 83.7–94.7) and 80% (95% CI 75.8–84.1), respectively. This difference was not statistically significant (Pv>0.05). Moreover, there was no association between the age of the mother and the presence of IgG among their offspring (Table 2).

**Table 2:** Risk indicators for the presence of immunoglobulin in the oral fluid from infants

	**Presence/absence of IgG in oral fluid at six months**		**Presence/absence of IgG in oral fluid before measles vaccination at nine months**		**Presence/absence of IgM & IgG in oral fluid after measles vaccination**	
**Variable**	***OR***	***95% CI***	***OR***	***95% CI***	***OR***	***95% CI***
						
Gender (F, M)	1.1	0.74–1.61	1.1	0.78–1.55	0.66	0.32–1.37
Breast feeding (Yes, No)	1.02	1–1.04	0.88	0.39–1.95	2.73	0.62–1.20
Measles history (Yes, No)	1.39	0.17–11.43	0.78	0.14–4.28	0.22	0.01–3.70
Type of birth (Normal, Caesarean)	0.94	0.81–1.83	1.18	0.58–2.39	0.54	0.12–2.51
Low-birth-weight (Yes, No)	1.03	0.25–4.21	0.78	0.44–1.36	0.36	0.13–1.94
Prematurity (Yes, No)	1.02	0.7–1.48	0.57	0.31–1.05	1.70	0.54–3.35
Weight for height cat (≥ -2, <-2)	-	-	0.78	0.32–1.87	1.18	0.12–1.01
Weight for age cat (≥ -2, <-2)	0.59	0.1–3.58	2.16	0.75–6.19	0.75	0.15–3.54
Height for age cat (≥ -2, <-2)	1.26	0.46–3.46	0.79	0.55–1.13	0.50	0.18–1.39
Mother's IgG (Pos, Neg*)	1.96	0.62–6.17	1.57	0.86–2.87	1.62	0.59–3.48
Mother's age ≤ 28 y, ≥ 29 y	0.81	0.29–2.32	1.15	0.61–2.19	1.08	0.67–1.75

OR – Odds Ratio; Pv – chi-square P-value; F – female; M – male; IgG – Immunoglobulin type G; IgM – Immunoglobulin type M; Pos – Positive; Neg – Negative.

#### Nutritional status

A great majority of the children enrolled in this study were still breastfeeding (97.0%; 95% CI 95.2–98.2). The mean birth weight was 3.064 grams (SD 450, range 1.900–4.400)

No children with clinical malnutrition were observed. Among the 211 infants aged six-months, 181 (85.7%; 95% CI 80.4–89.8), 185 (87.6%; 95% CI 82.5–91.4) and 151 (71.5%; 95% CI 65.1–77.2) children had more than minus two Z-score for weight-for-height category, weight-for-age category and height-for-age category, respectively. Among the 301 infants aged nine-months, 219 (72.7%; 95% CI 67.4–77.4), 226 (75.0%; 95% CI 69.9–79.6) and 186 (61.7%; 95% CI 56.1–67.1) children had more than minus two Z score for weight-for-height category, weight-for-age category and height-for-age category, respectively.

We did not find any association between nutritional parameters and the presence of measles-specific IgG in six- and nine-month-old children (Table 2).

#### Premature and low-birth-weight children

Only about a third (36.5%; 95% CI 32.4–40.7) of the mothers presented their last Pregnancy-Monitoring Card. Based on card records, 127 (67.9%) of the babies had been born at less than 37 weeks of gestation. All 512 children had a Road-to-Health Card, but only 442 (86.3%; 95% CI 82.9–89.1) of the cards had the birth weight recorded. Among 442 birth weight records, 58 (13.1%; 95% CI 10.1–16.7) indicated a child born with less than 2,500 grams.

We did not find any association between premature or low-weight births and the presence of measles-specific IgG in six- and nine-month-old children (Table 2).

### Vaccine seroconversion

In a group of 198 nine-month-old children that returned to the follow-up visit after vaccination, 52 were excluded from this analysis because they were already positive for IgG before vaccination. Among the remaining 146 infants, four provided insufficient oral fluid volumes to test for both antibody isotypes; we choose to test these samples only for IgM antibody. Only 85 (58.2%; 95% CI 50.2–66.2) children had a positive test for measles-specific IgM while 37 (26.0%; 95% CI 18.8–33.2) became positive for IgG. Among the 142 children tested for both isotypes, 19 were positive for both IgG and IgM and 20 tested negative for both. Therefore, in this study, the seroconversion rate after measles immunization was 84.2%.

Gender, mother's age (categorically divided into those born before and those after the introduction of EPI), breast-feeding, history of measles, type of birth, low-birth-weight, mother's immunity against measles (presence of IgG antibody) and nutritional status in bivariate analysis were not significantly associated with the presence of measles-specific IgG/IgM antibodies after vaccination (Table 2).

## Discussion

Measles vaccination has proved to be an extremely successful public health intervention and has already resulted in the elimination of measles in selected areas of the globe [2,22]. However, the success of measles immunization depends on many factors, including the absence of maternal antibodies at the time of vaccination [23]. Maternal IgG is transferred via the placenta during the last trimester of pregnancy and gradually wanes during first year of life [23]. In many SSA countries measles immunization is given at nine-months of age to avoid the interference of passively acquired IgG.

In our study, 82.4% of the children aged six-months did not have detectable levels of measles IgG in oral fluid. It is possible that a proportion of these infants had low levels of antibody only detectable in serum or by sensitive assays such as the plaque reduction neutralization [24,25]. Still, the oral fluid test used in our study identifies individuals with protective levels of IgG [21]. In periods of wild virus circulation, children identified as IgG-negative in this study would be susceptible to measles for at least three months before reaching the accepted age for vaccination.

In the period ranging from September 2004 (when the oldest infant included in this study was born) and the end of the study in September 2005, only sporadic measles cases (41 and 53 reported cases in 2004 and 2005, respectively) and no outbreak was observed in the study area *(Weekly Epidemiologic Bulletin, MoH Mozambique)*. However, a recent assessment of the routine surveillance system in Maputo City has shown significant under-reporting of measles [4]. In our study, measles virus circulation among infants younger than six-months was demonstrated in our setting by two-fold evidence: firstly, two infants presented with measles specific IgM in oral fluid and, secondly, a small proportion of children with measles specific IgG in oral fluid were born to mothers that did not have detectable levels of measles IgG. However, as none of the mothers recalled clinical episodes of measles-like illnesses in their children, the exposure to wild-virus probably only resulted in sub-clinical infections [26,27]. Further indirect evidence for sub-clinical measles in infants comes from the fact that measles specific IgG was present in a significantly higher proportion of nine-month-old children when compared to six-month-old children (30.5% vs. 12.3%, p < 0.001). In fact, recent exposure to measles in some IgG-positive children was self-evident due to the simultaneous presence of measles-specific IgM. However, we can not exclude the possibility of false positive test reactions among the nine-month cohort. The impact of sub-clinical measles on the health of unvaccinated young children or on vaccine-induced immunity remains to be determined.

Premature birth, low-birth-weight and malnutrition were not associated with the presence of immunoglobulin in oral fluid from infants (Table 2). We found a relatively high proportion of children with less than minus two z-scores on nutrition parameters. It has been previously documented that international nutritional and growth evaluation scales may not be appropriate for recourse-poor, tropical areas of the world [28]. Mounting evidence points out that this apparent state of reduce growth does not affect the physical activity of the individuals living in the tropics [28]. Nevertheless, the consequences of this growth and nutritional status on the physiology of the immune system require further research. Surprisingly, 68% of the children included in this study were born at less than 37 weeks of gestation. This information, based on the last menstrual cycle date provided by the mother, was collected from the Pregnancy-Monitoring Card and is known to be of low accuracy [29,30].

We expected that mothers aged 28 years and younger, believed to have vaccine-induced immunity, would give birth to offspring with a wider "window of susceptibility" than mothers with immunity from natural infection [31,32]. Two situations may explain why this is not the case in our setting (Table 2). First, periodic measles outbreaks in Maputo City may have provided opportunities for immunity boosters in all women and eliminated the classical picture of lower antibody levels in vaccinated women. We were not able to verify this finding because the oral fluid test for measles IgG is not a quantitative assay. Second, the IgG present in children may have resulted from exposure to wild-virus after complete waning of maternal IgG. The cross-sectional design of our study and the lack of laboratory assays that can differentiate between antibodies derived from natural infection as opposed to vaccination limited further investigation of these questions.

Success of vaccination at the individual level can be measured by the resulting immune response. We investigated seroconversion after immunization by measuring measles-specific IgG and IgM in oral fluid. In Maputo City, the cold chain is well monitored and follows the international WHO standards. Our study showed that 84.2% of the children developed an immune response with measles specific IgG and IgM detectable in oral fluid. While the use of more sensitive serum or plasma testing may have shown a slightly higher rate of seroconversion [33], the observed rate in our study can be considered as satisfactory when compared to the minimum acceptable seroconversion rate of 85% [34].

This study did not find any factor significantly associated with seroconversion after vaccination. We can not exclude the possibility that low levels of IgG, that were undetectable in oral fluid but that could modulate the immune response against the vaccine, played a role in determining the observed rate of seroconversion [35]. As some of the evidence presented in this study suggests, measles antibodies can arise from exposure to wild-virus among unprotected children. We did not investigate the role of HIV infection in the immune response of vaccinated children. It is possible that primary and secondary measles vaccine failures in HIV infected children play a role in maintaining the circulation of wild-type virus in Mozambique [36]. Although the impact of the HIV epidemic should be taken in consideration when updating measles immunization policy, it is unlikely that countries will adopt different measles immunization policies aimed at HIV-infected children.

The oral fluid tests for measles-specific IgG and IgM used in this study constitute a good alternative to serum/plasma assays for epidemiological surveys as invasive specimen collection is avoided and samples may be stored for up to 21 days at room temperature [16,21]. The main limitations of these tests are posed by difficulties in obtaining sufficient volumes of oral fluid from infants and by the relatively low sensitivity and specificity of the IgG assay. Indeed, it may be possible that the 12 IgG positive infants born to IgG negative mothers represent false-positive results. However, this is unlikely as five of these children were also IgM positive, further supporting our hypothesis that children are exposed to wild-type measles virus. It is also not likely that the higher prevalence of measles IgG in nine months old children is due to a higher rate of false-positive results in this age group.

Due to the simplicity and robustness of the oral fluid EIA, this is now routinely used in the MMR reference laboratory in the United Kingdom. In 2008, Vainio et al. [37] recommended the testing of oral fluid specimens using the MicroImmune assay for measles surveillance in Norway.

Measles virus circulation continues to occur in SSA even though many of countries have increased the routine vaccination coverage. An assessment of the surveillance system in Mozambique showed that in Maputo City during 1998 measles vaccine coverage was 85% and measles attack rate in children less than nine-months of age was about 24 per 10,000 inhabitants [4]. In three outbreaks studied in 1998 and 2002, children under nine-months of age accounted for between 11.1% and 22.2% of all reported measles cases [4], a proportion similar to the one observed in communities where immunization has not been introduced [38]. These epidemiological observations are in line with our biological findings that, in most children, passive immunity against measles wanes well before immunization takes place. Therefore, for countries like Mozambique, efforts to narrow the window of susceptibility should be regarded as important as those directed towards cutting the circulation of wild virus through increasing the coverage of routine and supplementary vaccination.

## Conclusion

The introduction of a fixed two-dose immunization schedule may be necessary if the window of susceptibility is to be reduced. One possibility is to vaccinate before nine-months of age and provided a second dose at a later age. The lower prevalence of measles IgG in six-month-old children living in Maputo City shows that these infants seem to be susceptible to measles three months before the recommended age of vaccination. This study enlighten some of the problems related to measles vaccination schedule but does not bring enough evidence to advise change of vaccination policy without further investigations.

The road to measles elimination in SSA is paved with several biological and non-biological determinants. While studies continue investigate relevant scientific questions and evaluate the cost-effectiveness and feasibility of adjustments in the measles immunization schedule, efforts should continue to focus on increasing the coverage of the EPI and introduction of supplementary vaccination.

## Competing interests

The authors declare that they have no competing interests.

## Authors' contributions

Jagrati Jani, Carol Holm-Hansen, Gunnar Bjune and Ilesh Jani conceptualized the study. Jagrati Jani, Tufária Mussá, Arlinda Zango, Ivan Manhica were responsible for data acquisition. All the authors were responsible for analysis and interpretation of data. IVJ provided technical input, review and project direction. All the authors collaboratively wrote the manuscript. All authors read and approved the final manuscript.

## Pre-publication history

The pre-publication history for this paper can be accessed here:


